# Blood plasma levels of biomarkers of liver status and lipid profile among nail technicians occupationally exposed to low-level mixture of volatile organic compounds

**DOI:** 10.1007/s00420-020-01599-2

**Published:** 2020-11-06

**Authors:** Peter Grešner, Magdalena Beata Król, Radosław Świercz, Jolanta Gromadzińska

**Affiliations:** 1 Department of Toxicology and Carcinogenesis, Nofer Institute of Occupational Medicine, 8, Sw. Teresy str., 91-348 Lodz, Poland; 2 Department of Biological and Environmental Monitoring, Nofer Institute of Occupational Medicine, 8, Sw. Teresy str., 91-348 Lodz, Poland

**Keywords:** Nail technician, VOC, Combined exposure, Transaminases, Cholesterol, HDL, Glucose

## Abstract

**Purpose:**

Nail technicians (NTs) are exposed to a low-level mixture of volatile organic solvents (VOCs), yet the health hazards related to such exposure are unknown. This study thus aimed to compare the blood plasma levels of selected biomarkers related to liver status and lipid profile among occupationally exposed NTs and unexposed controls. Associations between out-of-normal-range levels of such biomarkers and occupational exposure to VOCs mixture have also been investigated.

**Methods:**

The study enrolled 145 female NTs and 152 unexposed controls. Biochemical analyses were performed using spectrophotometric assays and obtained data were analyzed using general linear model and Poisson regression modelling adjusted to multiple confounders.

**Results:**

Compared to controls, NTs presented significantly increased plasma activities of ALT (2.04 ± 0.63 ln-U/l vs. 1.25 ± 0.71 ln-U/l; *p* < 0.0001) and AST (2.73 ± 0.25 ln-U/l vs. 2.08 ± 0.95 ln-U/l; *p* < 0.0001), and significantly increased plasma levels of TG (4.38 ± 0.53 ln-mg/dl vs. 4.21 ± 0.42 ln-mg/dl; *p* < 0.05) and TC/HDL ratio (1.18 ± 0.36 vs. 1.02 ± 0.27; *p* < 0.0005). Plasma levels of HDL were significantly lower among NTs (4.02 ± 0.29 ln-mg/dl vs. 4.21 ± 0.26 ln-mg/dl; *p* < 0.0001). Moreover, NTs were found to present significantly increased risk of occurrence of clinically relevant plasma HDL levels below 3.91 ln-mg/dl (i.e., 50 mg/dl; RR = 1.58, 95% CI 1.07–2.32, *p* < 0.05), as well as increased risk of clinically relevant TC/HDL ratio above the normal range limit of 3.5 (RR = 1.68, 95% CI 1.19–2.35, *p* < 0.005), as compared to unexposed controls.

**Conclusion:**

Nail technicians are subject to adverse changes in selected plasma biomarkers related to liver functions, some of which may be of clinical relevance.

## Introduction

Long-term occupational exposure to a low-level mixture of volatile organic compounds (VOCs) can be found in many occupational groups, including nail technicians. The number of nail salons and, consequently, the number of nail technicians has increased dramatically since the couple of last decades (Roelofs et al. 2008). According to unpublished data of the Central Statistical Office, it is estimated that there are more than 100,000 registered nail technicians in Poland. Nail technicians provide services which are unique in several specific aspects: contact with many toxic and potentially dangerous chemicals (acrylates, organic solvents, formaldehyde or sulfonamide–formaldehyde resins), work in small and poorly ventilated rooms, frequent overtimes, procedures requiring the use of chemicals close to technician’s breathing zone and eyes.

Since the concentrations of chemicals used in this industry are very low, health hazards related to occupational exposure in this workgroup are still underestimated. Meanwhile, the vast majority of nail technicians are significantly concerned about their health status, in line with which are the numerous reports providing evidence on increased occurrence of various non-specific adverse health effects possibly associated with occupational environment, including skin, eyes and upper airways mucosal irritations and inflammations, headaches, bronchial asthma, but also musculoskeletal and reproductive system disorders and limited results of pulmonal function tests (Lazarov 2007; Roelofs et al. 2008; Quach et al. 2008, 2011; Reutman et al. 2009; Harris-Roberts et al. 2011; Pak et al. 2013; Park et al. 2014). In addition to these, we have recently reported that when compared to office-based non-exposed controls, nail technicians show an earlier onset of the majority of above-mentioned non-specific symptoms, which they subjectively attribute to work environment (Grešner et al. 2017). Moreover, nail technicians seem to be subject to general deregulation of selected biomarkers of oxidative stress and DNA damage with distinct seasonal variability (Grešner et al. 2016), correlating with airborne ethanol levels in the workplace (Grešner et al. 2015).

Liver is considered the main site of VOCs detoxification. Liver damage, supposedly induced mainly due to toxic action of their intermediates and metabolites produced by the liver itself, has to be thus considered among adverse health effects caused by occupational exposure to VOCs (Malaguarnera et al. 2012). Pathophysiologic mechanism of such hepatotoxicity is still under investigation; nevertheless, it is believed to be characterized by organic and functional damage of the liver resulting from ongoing inflammation, dysfunction of cytochrome P450 enzyme system, mitochondrial dysfunction, and oxidative stress. All these jointly contribute to disruption of hepatocytes and their plasma membranes, altered function of transport proteins, cytotoxic T-cell activation, triggering of hepatocytes’ apoptosis and bile duct injury. Such conditions are clinically manifested by fatigue, loss of appetite, arthralgia, hyperstransaminasemia, splenomegaly, liver steatosis and necrosis (Brautbar and Williams 2002; Malaguarnera et al. 2012). Although hepatotoxic effects of certain organic solvents (including ethanol, toluene, xylene or chloroform) have long been studied, health and hepatotoxic effects of a long-term exposure to low levels of a mixture of organic solvents together with other VOCs, a setting which is the most frequent in the case of occupational exposure, have only been poorly investigated or remain undetermined at all. In this context, it is important to note that prolonged occupational exposure to low levels of VOCs mixture can be found in several other workgroups in contact with paints, lacquers, photographic chemicals, as well as in production of plastics and synthetic rubber (Mechilli et al. 2008; Malaguarnera et al. 2012).

Therefore, here we report a study in which we analyzed the blood plasma levels of selected biochemical markers related to liver functions and status, including blood plasma activities of alanine (ALT) and aspartate (AST) transaminases, total plasma level of cholesterol (TC), blood plasma levels of high density lipoproteins (HDL), low density lipoproteins (LDL), and triglycerides (TG), plasma TC/HDL ratio as well as fasting plasma glucose level (fGlu) among nail technicians with long-term low-level exposure to a mixture of VOCs and compared these levels to those obtained in unexposed office-working control subjects. Furthermore, we tried to compare the risk of occurrence of out-of-normal-range levels of these biomarkers between the two groups enrolled in the study.

## Methods

### Study and control subjects

Details on study and control groups used in this study have already been published (Grešner et al. 2015). Briefly, a total of 318 nail and/or beauty salons providing manicure and/or artificial nail sculpturing services within various parts of the city of Lodz in Central Poland were contacted during the recruitment to take part in the study. Out of these, 109 salons responded, from which the study group consisting of 145 female nail technicians [aged 21–64 years; median age: 34 years, interquartile range (IQR): 27–42 years], occupationally exposed to low-levels of VOCs, was ultimately created. All nail technicians declared that they had not been performing any other job previously. Median length of the period of employment among nail technicians was 6 years (IQR: 1–39 years).

The control group consisted of female subjects drawn from the general population of the city of Lodz, Central Poland, meeting the main inclusion criteria, i.e., (a) not being involved in the nail industry (currently nor previously), (b) spending most of the work-time in a sitting position, similar to nail technicians, and (c) not being occupationally exposed to VOCs (even in the past). Since the study group consisted solely of women, male subjects were not considered for participation in the control group, either. The control group thus consisted of 152 women (aged 19–59 years; median age: 33 years, IQR: 27–42 years) and was comprised of 107 (70%) general office and 45 (30%) academic workers without any current nor previous occupational contact with VOCs. The lack of any history of occupational exposure to VOCs among controls was determined based on a simple custom-made questionnaire. Median length of the period of employment among control subjects was 7 years (IQR: 0.1–42 years).

All study and control subjects were of Caucasian descent and were recruited within 2011–2012. Environmental exposure to VOCs was not considered in this study and was assumed to be similar in both groups. All samplings were scheduled to ensure that all subjects were healthy and free of any health-related problems (such as fever or any infections). Each participant of the study fulfilled a short researcher-made questionnaire designed to collect supplementary information on tobacco smoking and alcohol ingestion. Subjects were considered non-smokers or current-smokers according to the US Centers for Disease Control and Prevention definitions (Schoenborn and Adams 2013). Former (> 5 years since smoking cessation) and never-smokers were both considered non-smokers. Current smokers were further characterized by current tobacco load expressed in pack-years (PY), i.e., the number of cigarette packs smoked par day × the number of years lived smoking. Current tobacco load for non-smokers was assumed to be zero. Average weekly ethanol (etOH) ingestion was estimated based on self-declared number of average weekly consumption of alcoholic drinks and beverages, assuming a typical amount of 25 ml, 12.5 ml and 20 ml etOH per bottle of beer, glass of wine and a drink shot, respectively. Basic characteristics of both groups can be found in Table 1, complete description thereof is provided elsewhere (Grešner et al. 2015).

**Table 1:** Basic characteristics and levels of analyzed blood plasma biomarkers among nail technicians and unexposed control subjects

		Controls (*n* = 152)			Nail technicians (*n* = 145)		
		Median	IQR	Range	Median	IQR	Range
Age (years)		33	27–42	19–59	34	27–42	21–64
BMI		22.3	20.5–24.3	17.2–36.7	22.6	20.0–25.6	8.6–37.1
Current tobacco load (pack-years)^a^		10.0	2.5–15.0	0.1–25.0	3.1	0.4–7.4	0.1–22.5
Ethanol ingestion (ml/week)^a^		32.5	12.5–55.0	1.6–225.0	42.5	25.0–75.0	3.1–162.5
Σ(*C*_*i*_/*N*_*i*_)		–	–	–	0.033	0.014–0.081	0.002–0.333

	Normal range^e^	Mean	SD	Range	Mean	SD	Range
ALT (ln-U/l)^b^	< 3.53	1.25	0.71	0.56–3.18	2.04	0.63	0.56–3.50
AST (ln-U/l)^b^	< 3.53	2.08	0.95	0.56–3.26	2.73	0.25	0.56–3.55
TC (ln-mg/dl)	< 5.30	5.24	0.21	4.77–5.71	5.21	0.23	4.66–6.07
HDL (ln-mg/dl)^b^	> 3.91	4.21	0.26	3.40–5.08	4.02	0.29	3.04–4.86
LDL (ln-mg/dl)	< 4.61	4.62	0.37	3.13–5.34	4.61	0.49	2.12–5.89
TG (ln-mg/dl)^c^	< 5.01	4.21	0.42	2.46–6.09	4.38	0.53	3.18–6.47
ln(TC/HDL)^d^	< 1.25	1.02	0.27	0.31–2.02	1.18	0.36	0.18–2.26
fGlu (ln-mg/dl)	< 4.61	4.47	0.12	4.14–4.92	4.44	0.15	4.04–4.83

Basic characteristics of the study and control groups together with the levels of selected blood plasma biomarkers analyzed in the study. Basic characteristics were tested for between group differences using the Mann–Whitney *U* test. For blood plasma biomarkers, levels adjusted to differences in age, BMI, current tobacco load and weekly ethanol ingestion are shown, and statistical significance was inferred using the general linear model and further confirmed by the Schaffé post-hoc test

*BMI* body mass index, *IQR* interquartile range, *SD* standard deviation, *Σ(C*_*i*_*/N*_*i*_*)* ACGIH estimate of combined exposure to a mixture of VOCs

^a^*p* < 0.05

^b^*p* < 0.0001

^c^*p* < 0.05

^d^*p* < 0.0005

^e^Normal ranges for investigated biomarkers used in this study

Prior to any further measurements, written and informed consent for participation in this study was obtained from each participant. The study was performed under the guidelines of the Helsinki Declaration for human research and was approved by the Bioethics Committee of the Nofer Institute of Occupational Medicine (resolution no. 5/2011).

### Blood withdrawal

Blood withdrawal was performed always in the morning before work, at around 8:00–9:00 am, following a 12-h overnight fast. A sample of peripheral blood was collected from each participant involved in the study by venipuncture into tubes containing heparin as anticoagulants. Heparinized blood was centrifuged (10 min, 1500 × g, 4 °C) to isolate blood plasma.

Blood plasma was then frozen at − 80 °C and subsequently used to determine the blood plasma activities of AST and ALT, plasma levels of TC, HDL, LDL, TG and fGlu.

### Biochemical assays

Plasma activities of ALT and AST were determined spectrophotometrically using commercially available assay kits (Alpha Diagnostics, Poland) based on methods described by Wróblewski and LaDue (1956) and Karmen et al. (1955), respectively. TC, HDL, LDL and TG were analyzed spectrophotometrically in blood plasma using respective commercially available assay kits (Cholesterol Całkowity DST, HDL Cholesterol-Bezpośredni, LDL Cholesterol-Bezpośredni, Trójglycerydy DST, respectively; all from Alpha Diagnostics, Poland), according to well-established analytical methods (McGowan et al. 1983; Artiss and Zak 1997; Bachorik 1997; Xiao 2002). Fasting plasma glucose level was measured spectrophotometrically according to method described by Trinder (1969).

All spectrophotometric assays were performed using the Thermo Fisher Evolution 300 UV/VIS spectrometer (Thermo Fisher Scientific, USA).

### Air sampling and assessment of occupational exposure to VOCs

Relevant information on air sampling and assessment of occupational exposure of nail technicians to VOCs, performed according to method described previously by Gjolstad et al. (2006) with minor modifications, has been provided in all necessary details previously (Grešner et al. 2015). In this study, the assessment of occupational exposure to VOCs (including ethanol, acetone, toluene, 2-propanol, 2-butanone, ethyl acetate, isopropyl acetate, *n*-butyl acetate, hexamethyldisiloxane, methyl methacrylate and ethyl methacrylate) based on the combined exposure to a mixture of VOCs calculated according to the American Conference of Governmental Industrial Hygienists (ACGIH) formula:

$$\sum {\frac{{C_{i} }}{{N_{i} }} = \frac{{C_{1} }}{{N_{1} }}} + \frac{{C_{2} }}{{N_{2} }} + ... + \frac{{C_{n} }}{{N_{n} }},$$

where *C*_*i*_ and *N*_*i*_ refer to measured airborne concentration and time-weighted average occupational exposure limit (TWA-OEL) for *i*th out of *n* components, respectively, was used and is briefly summarized in Table 1.

### Statistical analyses

Normal distribution of numerical data was tested using the Shapiro–Wilk *W* test. Since the vast majority of raw data failed to follow the normal distribution, natural-base logarithm data transformation was used prior to any statistical analyses. Logarithmically transformed data following normal distribution are presented as mean with corresponding standard deviation (SD) and expressed in respective ln-units. Analysis of outliers was performed based on estimated Cook’s distances. Basic inter-group comparisons of normally distributed ln-transformed data adjusted for differences in age, body mass index (BMI), current tobacco load (expressed in PY), average weekly etOH ingestion (expressed in ml per week) and combined occupational exposure to a mixture of VOCs [i.e., Σ(*C*_*i*_/*N*_*i*_)] were performed using the general linear model (GLM) involving the five above-mentioned factors as confounders possibly affecting the compared parameters.

Plasma levels of all investigated biomarkers were then dichotomized with respect to respective normal range limit values and then the proportion of subjects with adverse levels of respective biomarkers (i.e., levels above respective upper limit values for ALT, AST, TC, LDL, TG, TC/HDL and fGlu; levels below the lower limit value for HDL) were tested for statistically significant between-group differences using the Poisson regression modelling adjusted to differences in age, BMI, current tobacco load, average weekly etOH ingestion and combined occupational exposure to a mixture of VOCs [i.e., Σ(*C*_*i*_/*N*_*i*_)]. As a result, the group of nail technicians was characterized by relative risk (RR) estimates (with corresponding 95% confidence intervals; 95% CI), indicating the fold-change in the risk of the occurrence of adverse levels of respective biomarkers when compared to controls.

All statistical analyses were performed using the Statistica 10 software (StatSoft, Tulsa, OK, USA).

## Results

### Blood plasma levels of selected biochemical markers among nail technicians and controls

Results of biochemical assays together with respective normal ranges are shown in Table 1. Only ALT activities were found to fit within normal ranges in both groups of subjects, while only two nail technicians showed AST levels slightly exceeding the upper limit value. Plasma levels of TC, HDL, LDL, TG, TC/HDL and fGlu, were found to span beyond those ranges in both groups, with values observed among nail technicians reaching much farther beyond the respective normal range limits.

Following adjustment to age, BMI, current tobacco load, average weekly etOH ingestion and combined exposure to VOC mixture [Σ(*C*_*i*_/*N*_*i*_)], nail technicians were shown to present a significantly increased blood plasma activities of ALT (NT vs. controls: 2.04 ± 0.63 ln-U/l vs. 1.25 ± 0.71 ln-U/l; *p* < 0.0001) and AST (NT vs. controls: 2.73 ± 0.25 ln-U/l vs. 2.08 ± 0.95 ln-U/l; *p* < 0.0001) compared to controls. Statistically significant differences between the two groups were found also for blood plasma levels of HDL (NT vs. controls: 4.02 ± 0.29 ln-mg/dl vs. 4.21 ± 0.26 ln-mg/dl; *p* < 0.0001) and TG (NT vs. controls: 4.38 ± 0.53 ln-mg/dl vs. 4.21 ± 0.42 ln-mg/dl; *p* < 0.05) which were found to be significantly decreased and increased, respectively, among nail technicians. Since the level of TC did not vary significantly between the groups (NT vs. controls: 5.21 ± 0.23 ln-mg/dl vs. 5.24 ± 0.21 ln-mg/dl; NS), the resulting plasma TC/HDL ratio was found to be significantly increased among nail technicians (NT vs. controls: 1.18 ± 0.36 vs. 1.02 ± 0.27; *p* < 0.0005). No statistically significant inter-group differences were found for plasma levels of LDL and fGlu.

### The risk of occurrence of out-of-normal range biomarker levels among nail technicians

As summarized in Table 2, the Poisson regression modelling adjusted to differences in age, BMI, current tobacco load, average weekly etOH ingestion and combined occupational exposure to a mixture of VOCs (i.e., Σ(*C*_*i*_/*N*_*i*_); for levels of confounders, see Table 1) revealed increased risk of out-of-normal-range plasma HDL and TC/HDL levels among occupationally exposed nail technicians when compared to unexposed controls.

**Table 2:** Risk of incidence of plasma biomarker levels falling beyond normal plasma ranges—results of Poisson regression modelling

	*β* [95% CI]	RR [95% CI]
ALT [> 3.53 ln-U/l]	– [n.a.–n.a.]	– [n.a.–n.a.]
AST [> 3.53 ln-U/l]	– [n.a.–n.a.]	– [n.a.–n.a.]
TC [> 5.30 ln-mg/dl]	0.07 [− 0.21–0.35]	1.08 [0.81–1.43]
HDL [< 3.91 ln-mg/dl]^a^	0.46 [0.07–0.84]	1.58 [1.07–2.32]
LDL [> 4.61 ln-mg/dl]	0.19 [− 0.05–0.42]	1.21 [0.95–1.53]
TG [> 5.01 ln-mg/dl]	0.61 [− 0.12–1.33]	1.83 [0.89–3.80]
ln(TC/HDL) [> 1.25]^b^	0.52 [0.18–0.85]	1.68 [1.19–2.35]
fGlu [> 4.61 ln-mg/dl]	0.39 [− 0.18–0.97]	1.48 [0.84–2.63]

Presented are the respective regression coefficients (*β*) and resulting relative risks (RR) with corresponding 95% confidence intervals (95% CI) obtained using Poisson regression modelling adjusted to age, BMI, current tobacco load, weekly ethanol ingestion, and combined occupational exposure to a mixture of VOCs [i.e., Σ(*C*_*i*_/*N*_*i*_)], employed to compare the risk of incidence of levels of analyzed biomarkers falling beyond the respective normal ranges in the two groups analyzed in the study. Criterion for a biomarker level being considered as falling beyond the respective normal range is provided next to each biomarker name. RR values reflect the relative risk increase in the group of nail technicians with respect to the risk among controls

^a^*p* < 0.05

^b^*p* <  0.005

Among nail technicians, the risk of incidence of plasma HDL levels falling below normal range limit (i.e., 3.91 ln-mg/dl equal to 50 mg/dl) was higher by some 58% (RR = 1.58, 95% CI 1.07–2.32, *p* < 0.05), while in the case of plasma TC/HDL ratio the risk of its level exceeding the normal range limit (i.e., 1.25 in ln-units, 3.5 in linear units) was higher by approximately 68% (RR = 1.68, 95% CI 1.19–2.35, *p* < 0.005), as compared to unexposed controls. No statistically significant risk changes were observed for any other plasma biomarker investigated in this study (see Table 2).

## Discussion

The hereby presented study shows that nail technicians, a workgroup with long-term occupational exposure to low-level mixture of VOCs, are subject to significantly increased blood plasma levels of ALT and AST activities, plasma levels of TG and TC/HDL ratio, as well as to significantly decreased plasma level of HDL. While ALT and AST activities fell within the physiological range in nearly all controls and nail technicians, TG, TC/HDL and HDL (but also TC, LDL, and fGlu) span beyond the respective physiological ranges, with much pronounced divergence from this range among nail technicians. It is worth noting that these outcomes, adjusted to several known confounders (age, BMI, tobacco load, ethanol ingestion), were found in a workgroup with relatively low, yet very specific type of occupational exposure, where neither TWA-OEL values of monitored substances (Grešner et al. 2015) or the respective ACGIH threshold for combined exposure were found to be exceeded (median and maximum levels of 3.3% and 33.3% of the ACGIH threshold, respectively). It only adds to an increasing amount of evidence suggesting that application of occupational exposure limits normally used in other industrial branches may be inappropriate in the case of nail care industry. The assessment of occupational exposure based solely on low concentrations of used chemicals, not accounting for other aspects of work procedures (closed rooms, short distance between working zone and airways, etc.) may in fact lead to significant underestimation of occupational exposure-related health hazards this specific workgroup is exposed to (Roelofs and Do 2012).

A number of studies reporting altered levels of biomarkers of liver status (plasma activities of ALT, AST, alkaline phosphatase, *γ*-glutamyl transferase) as well as altered blood plasma lipid profile (increased plasma levels of TC, TG, and LDL) as a result of an exposure to a mixture of VOCs under various occupational settings can already be found in the literature (Brodkin et al. 2001; Brautbar and Williams 2002; Kaukiainen et al. 2004; Attarchi et al. 2007; Liu et al. 2009; Mohammadi et al. 2010; Salehpour et al. 2019). However, all these studies are related to heavy industrial environment in which the concentrations of used chemicals are considerably higher than those used in nail care industry. Even though the number of reports related to very specific long-term low-level occupational exposure of nail technicians seems to grow rapidly in recent years [for example, (Hadei et al. 2018; Ceballos et al. 2019; Zhong et al. 2019; Moradi et al. 2019; Lamplugh et al. 2019; Craig et al. 2019)], they are mainly focused on environmental and biological monitoring of such exposure. The literature concerning biochemical effects of such exposure, on the other hand, is still relatively scarce (Yoon et al. 2010; Grešner et al. 2015, 2016). Thus, the apparent lack of interest of scientific community in multi-faceted understanding of health hazards nail technicians are exposed to in their work environment seems to be of concern, especially given the number of previous studies related to various clinically manifested or subjectively reported disorders (Lazarov 2007; Roelofs et al. 2008; Quach et al. 2008, 2011; Reutman et al. 2009; Harris-Roberts et al. 2011; Pak et al. 2013; Park et al. 2014).

In the hereby presented study we also aimed to investigate whether the alterations in selected plasma levels of biomarkers related to liver functions and status observed among occupationally exposed nail technicians can be of any clinical significance. The two groups enrolled in the study were thus compared in terms of the incidence of out-of-normal-range plasma levels of analyzed biomarkers by means of the Poisson regression modelling adjusted to several known confounders. The regression modelling revealed that nail technicians are indeed a subject to increased risk of clinically significant low plasma levels of HDL (i.e., those falling below the plasma normal range limit of 50 mg/dl), with the risk increment of almost 60% as compared to controls. Similar increased risk of clinically significant high plasma ratio of TC/HDL (i.e., values exceeding the plasma normal range limit of 3.5), with the respective risk among nail technicians being increased by almost 70%, as compared to unexposed controls, was also found. It must be emphasized that such changes often are considered as indication of preclinical states leading to many current civilization diseases including metabolic syndrome, diabetes or cardiovascular diseases, and might thus be of clinical relevance (Ko et al. 2015; Music et al. 2015; Kim and Han 2018).

Given the approximately equal median length of the period of employment among nail technicians and controls (6 years vs. 7 years, respectively), increased incidence of such adverse outcomes raises a question whether nail technicians might be subject to faster onset of clinically relevant alterations, at least in the case of some of the hereby investigated plasma biomarkers. Cox proportional hazard regression modelling, in which the length of the employment period of nail technicians was considered as time at risk, with right censoring based on the occurrence of plasma biomarker levels falling beyond the normal plasma range, revealed certain marginally significant (*p* ≈ 0.10) suggestions that the proportions of nail technicians with plasma levels of HDL and fGlu beyond respective normal ranges tend to rise faster over time under higher Σ(*C*_*i*_/*N*_*i*_) values. Of interest might especially by the plasma HDL, for which, in a separate Cox proportional hazards regression analysis with right-censoring based on HDL median value (4.11 ln-mg/dl, approx. 61 mg/dl), the association between the time of onset of plasma levels below median value and the level of occupational exposure to VOCs mixture became statistically significant (*p* < 0.01; data not shown). Even though the employment of Cox proportional hazard regression models in cross-sectional studies with retrospectively obtained time-at-risk data might not be common, such models were shown to provide more statistical power than, for example, logistic regression models, even if the “real” follow-up data are unavailable (van der Net et al. 2008). Nevertheless, there is one crucial assumption behind such approach, namely the model assumes that all analyzed risk factors are constant throughout the time at risk (van der Net et al. 2008). As we do not have any information on past levels of analyzed risk factors, confounders, as well as on exact time at risk after which the event of interest occurred, it is obvious that this requirement has not been met in this study. Therefore, hereby cited outcomes of Cox regression modelling need to be considered as presumably useful, yet still very uncertain indications of plausible associations between the time of onset of adverse biochemical alterations observed among nail technicians and the level of occupational exposure. Such indications might, however, prove valuable especially considering the very specific type of exposure in this workgroup and challenges related to ensuring constant levels of the above mentioned factors and confounders over a period of several to several tens of years, even if a proper follow-up period was planned. These difficulties might, probably, be among reasons why the number of studies accounting for the length of employment period (i.e., the time at risk) while analyzing adverse effects of occupational exposure among nail technicians are very limited so far (to date only Grešner et al. 2017; Reutman et al. 2009). Further research in this field seems thus to be warranted.

On the other hand, we ought to emphasize that due to very close age-match between the two groups, we were able to eliminate any possible bias introduced by known discrepancies in health status between young working population and the general one as well as the bias resulting from comparing the young group of nail technicians to general population (McDonald and Checkoway 2000). However, a certain degree of bias introduced by the fact that salons not meeting certain occupational hygiene criteria willingly refused to participate in the study, could not have been avoided.

To sum up, here we report that long-term exposure to a low-level VOCs mixture is associated with altered levels of plasma biomarkers related to liver status and plasma lipid profile, and that especially in the case of plasma HDL and TC/HDL levels, such exposure leads to 60–70% higher risk of the occurrence of clinically relevant levels out of normal blood plasma ranges. These changes seems to be in line with concerns expressed recently by the French Agency for Food, Environmental and Occupational Health and Safety (ANSES) which issued an official report (Anses 2017) in October 2017, stressing out that nail technicians are subject to several hundreds of various substances, 150 of which are of ‘very high concern’ or ‘concern’, and criticizing the ineffectiveness of commonly used protective measures and the lack of appropriate awareness of the hazards.

## Data Availability

All data are available from study authors upon request.
